# Implementing a system of quality-of-life diagnosis and therapy for breast cancer patients: results of an exploratory trial as a prerequisite for a subsequent RCT

**DOI:** 10.1038/sj.bjc.6604505

**Published:** 2008-07-29

**Authors:** M Klinkhammer-Schalke, M Koller, C Ehret, B Steinger, B Ernst, J C Wyatt, F Hofstädter, W Lorenz

**Affiliations:** 1 Tumor Center Regensburg e.V., Regensburg 93053, Germany; 2 Center for Clinical Trials, University Hospital Regensburg, Regensburg 93053, Germany; 3 Health Informatics Center, University of Dundee, Scotland DD2 4BT, UK

**Keywords:** quality of life, implementation, breast cancer, exploratory trial, patient–doctor agreement, positivity criterion

## Abstract

A system for quality-of-life diagnosis and therapy (QoL system) was implemented for breast cancer patients. The system fulfilled the criteria for complex interventions (Medical Research Council). Following theory and modeling, this study contains the exploratory trial as a next step before the randomised clinical trial (RCT) answering three questions: (1) Are there differences between implementation sample and general population? (2) Which amount and type of disagreement exist between patient and coordinating practitioners (CPs) in assessed global QoL? (3) Are there empirical reasons for a cutoff of 50 points discriminating between healthy and diseased QoL? Implementation was successful: 74% of CPs worked along the care pathway. However, CPs showed preferences for selecting patients with lower age and UICC prognostic staging. Patients and CPs disagreed considerably in values of global QoL, despite education in QoL assessment by outreach visits, opinion leaders and CME: Zero values of QoL were only expressed by patients. Finally, the cutoff of 50 points was supported by the relationship between QoL in single items and global QoL: no patients with values above 50 dropped global QoL below 50, but values below 50 and especially at 0 points in single items, induced a dramatic fall of global QoL down to below 50. The exploratory trial was important for defining the complex intervention in the definitive RCT: control for age and prognostic stage grading, support for a QoL unit combining patient's and CP's assessment of QoL and support for the 50-point cutoff criterion between healthy and diseased QoL.

Bio-psycho-social medicine as an integrated approach has become a paradigm in western societies to understand illness (White, 2005) and achieve more complete and effective treatment (Wulff and Gotzsche, 2000; Gimmler *et al*, 2002). It is, however, complicated to get evidence for the ‘active ingredients’ (MRC Health Services and Public Health Research Board, 2000) of such therapeutic regimens, particularly when the outcome is not just survival, but relates to patient-reported end points such as quality of life (QoL) (Lorenz and Koller, 2002). Delivering the multifaceted ingredients of bio-psycho-social medicine requires complex structures of care, which have to be set up through systematic implementation strategies (Klinkhammer-Schalke *et al*, 2008).

The Medical Research Council (MRC) has termed such integrated approaches as ‘complex interventions’ (MRC Health Services and Public Health Research Board, 2000): they differ from drug *vs* placebo evaluations in classical randomised trials. Complex interventions are defined as those in which it is difficult to characterise precisely the ‘active ingredients’ of intervention and how they relate to each other. Studies must clarify these ingredients and relationships before the definitive randomised trial is conducted. MRC proposed a new procedure for introducing and evaluating complex clinical interventions including five phases: theory, modeling, exploratory trial, definitive randomised trial and long-term implementation. Each phase needs separate studies and study reports.

The present project introduces and evaluates a system of diagnosis and therapy of impaired QoL in cancer patients (QoL system) and adopted the MRC complex intervention approach. The first paper summarised the conceptual basis underlying the project (preclinical phase) and modelling (phase 1 of the MRC scheme, Table 1 (Klinkhammer-Schalke *et al*, 2008)).

The second paper addresses phase 2, the exploratory trial.

The goals of this trial were twofold: to introduce the concept of QoL to coordinating practitioners (CPs) in an epidemiologically defined region (implementation), and to generate data that allow to fine-tune the study protocol for a subsequent randomised clinical trial (RCT), answering three questions:

1. Did the CPs, during implementation, select a sample of patients, which was different from the general population? Were age and prognosis prominent factors for inclusion or exclusion?
2. To what extent and how specifically did patients' self-reports and doctors' judgements disagree about QoL?
3. Are there empirical reasons supporting the 50-point cutoff criterion in the QoL profile to discriminate between healthy and diseased QoL?

## Patients and methods

### Study design

According to small area analysis (Wennberg and Gittelson, 1973), a defined area in Bavaria was selected for implementing and evaluating the QoL system in breast cancer patients. Within the service area of the Tumor Center Regensburg it comprised, namely the urban county of Regensburg and the rural county of Amberg, in total 500 000 inhabitants. The project started with setting up the infrastructure and physician training in QoL methodology in August 2002. Patient recruitment started in December 2002 and was completed in December 2004.

### Study participants

The study included 170 patients with primary breast cancer, 10 clinicians (two clinicians from each of the five hospitals in the two areas), 38 CPs, 12 opinion leaders (i.e., influential physicians in the region, who are respected and were selected by the CPs) and 75 professional therapists providing the QoL therapeutic options, and the QoL unit with five experts, including two study coordinators and two data managers. The interaction and the relative responsibilities of these individuals were determined in a care pathway (Klinkhammer-Schalke *et al*, 2008) (Table 1).

Main patient inclusion criteria were diagnosis of primary breast cancer and informed consent to participate in the study. The study protocol was approved by the ethics committee of the University of Regensburg. No restrictions were made regarding the date of the breast cancer diagnosis or comorbidity, and there was no upper age limit. However, patients were excluded if they were less than 18 years, unwilling to participate in the study, or unable to self-complete the German version of the questionnaire because of their mental or physical condition or poor command of the German language.

Coordinating practitioners (CPs) were defined as those who cared for breast cancer patients before and after hospital stay. They were either gynaecologists or oncologists, or family doctors. CPs were eligible for the project if they gave their informed consent, had at least a moderate routine of caring for breast cancer patients (at least three patients in the years 1999–2001), and agreed to participate in the multifaceted implementation procedure including outreach visits, opinion leaders and continuing medical education in a quality circle. CPs were expected to recruit three to six patients for the study.

Professional therapists were those who provided QoL-enhancing therapies such as pain therapy, physiotherapy and lymphatic drainage, sports activities and nutrition counseling or social counseling (Table 1). Their professional background required specific training in the area of expertise. Professional therapists participated in quality circles of the Tumor Center Regensburg, that provided regular trainings using PDCA cycles (PDCA=**p**lan, **d**o, **c**heck and **a**ct) (Klinkhammer-Schalke *et al*, 2008).

### QoL profile and expert report

**Figure 1:**
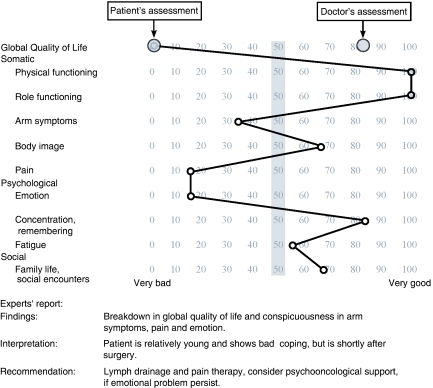
QoL profile and experts' report produced from the EORTC questionnaire by the patient and health status questionnaire by the doctor in the QoL unit and sent to the coordinating practitioner of the patient implemented in QoL diagnosis and therapy. Example of the largest difference between patient's and clinician's assessment of global QoL in 170 patients. Female patient with primary breast cancer, no. 170 in the series, 1 month after BCT with axillary lymph adenectomy, 44 years, married, two children. Prognostic classification T1c, N0, M0, G2, ER pos/PR pos, HER2neu neg. Cutoff level: 50 points (grey bar). For further details of the QoL system see reference Klinkhammer-Schalke *et al*, 2008.

The patient filled out QoL questionnaires (EORTC QLQ–C 30, version 2.0, plus the breast cancer module QLQ–BR 23) in their doctor's practice. Concurrently, the doctor filled out a health status form that included diagnostic, therapeutic and social information and a judgement of patient's QoL (Table 1). Both forms were sent to the QoL unit. Patients' QoL responses were transformed into a QoL profile (Figure 1) using a computerised QoL visualisation programme (Middeke *et al*, 2004). This profile was handed out to the experts in the QoL unit who independently formulated QoL diagnosis and treatment recommendations. These individual statements were discussed weekly in consensus meetings of the experts resulting in a group decision expert report. It was sent to the CP caring for the patient. It was ultimately the CP's choice to follow the recommendation, to initiate proper treatment by him/herself or to refer the patient to a professional therapist.

### Data management and statistical analysis

Patients' QoL questionnaire and doctors' health status questionnaire were collected as paper copies. Data were stored in a Microsoft ACCESS (version 2003) database. The QoL profile was produced using a specifically designed QoL-profiler programme (Middeke *et al*, 2004).

The statistical analyses of this implementation trial were primarily descriptive, using counts, percentages, means and standard deviations, medians and interquartile ranges, and 95% confidence intervals. Patient–doctor agreement was calculated using Spearman ρ correlation, interrater correlation coefficient (Shrout and Ford, 1979), and Bland–Altman plots (Bland and Altman, 1986). Group differences were calculated using the *χ*^2^-test, *t*-test, or Wilcoxon test where appropriate. All analyses were performed using SPSS for Windows, version 15.0.

## Results

### Did the coordinating practitioner select a sample of patients that was different from general population? Were age and prognosis prominent factors for inclusion or exclusion?

Key indicators for a successful implementation were the number of patients recruited by CPs and whether the sample was representative of the population of interest. Twenty-eight of 38 CPs were active in patient recruitment; this 74% compliance was satisfactory when compared with the 40% adherence rate in clinical guideline implementation (Margolis and Cretin, 1999). The majority of CPs recruited 3–6 patients, the median being 3. There were two outliers with 23 and 46 recruited patients, respectively.

We analysed the characteristics of the selected sample of patients (*n*=170) to identify representativeness in comparison to the population of breast cancer patients in the area investigated between January 2003 and June 2004 (*N*=509). This was possible because of complete documentation of breast cancer patients from 1992 on at the regional Tumor Center. The characteristics of the patients selected by the CPs were remarkable (Table 2). The small number of patients receiving chemo- and radiotherapy at the time of the investigation was because of the timing of patient enrollment: 75 patients were included within the first month after surgery and only 20 patients in the first year thereafter.

**Figure 2:**
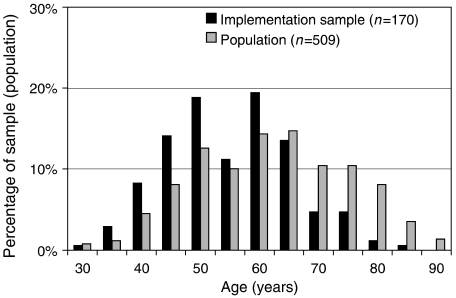
Age distribution of patients with breast cancer in the study region as documented by the tumour centre and that of patients selected by the coordinating practitioners during implementation. Histograms of the two groups (January 2003 until June 2004).

More importantly, there were differences between implementation sample and regional population regarding age. The frequency distribution was bell-shaped in the regional population, but skewed in the implementation sample, with patients missing above the age of 70 years (Figure 2). This became even clearer when proportions were compared. In the implementation sample only 18 out of 170 patients were ⩾70 years of age (11%; C.I. 6.8–16.1%), whereas in the regional population this proportion was almost three times larger, 137 out of 509 (27%, C.I. 22.9–30.6).

Comparisons were drawn with a historic cohort on primary breast cancer therapy in six regions of Germany, which was published in 2002 and comprised a sample of *N*=8661 (Engel *et al*, 2002). The proportion of patients aged ⩾70 years was 2425 out of 8661 (27.9%, C.I. 27.1–29.0), almost identical with the regional population.

**Figure 3:**
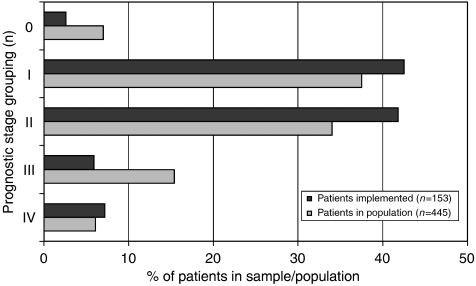
Prognostic stage (UICC) of the patients in the implementation sample and the regional population. Missing values in sample *n*=17, in population *n*=64, global test: *χ*^2^=14.689 (d.f. 4), *P*<0.005; single tests: UICC 0: *χ*^2^=3.913 (d.f. 1), *P*<0.05, UICC III: *χ*^2^=9.296 (d.f. 1) *P*<0.005, all other tests not significant.

The second attribute, which differed between implementation sample and population, was the prognostic stage (UICC; Figure 3). The CPs recruited a higher proportion of patients with the relatively favourable tumour stages 1 and 2, 87% of the cases, as compared with the 78% prevalence in the regional population (global *χ*^2^-test for all 5 stages: 14.7, d.f. 4, *P*<0.01), but the picture was more complex. The CPs selected fewer patients with carcinoma *in situ*, but more patients in the palliative group (for significances see legend in Figure 3).

### To what extent did patients' self-reports and doctors' judgements disagree?

It is well known that doctors and patients (Koller *et al*, 1996) show poor overlap in their assessment and judgement of clinical features. The more important question here was the extent and quality of disagreement after patients and doctors had been made familiar with the topic of QoL through an extensive implementation.

The most simple and often found method (Engel *et al*, 2002) for analysing differences is collapsing the individual data points into QoL scores and comparing their means. In our case, patient-reported global QoL was 66.7 (50.0–77.1) points (median and interquartile range), mean 61.5 and standard deviation 23.2, significantly lower than doctors' judgement of patients' global QoL, 75.0 (58.3–83.3), respectively, mean 71.0 and s.d. 18.8, nonparametric Wilcoxon test for paired data: *P*<0.001. To put this result into perspective, comparisons were drawn to the German Cancer Field Study (Kerr *et al*, 2003) and the reference values of a non-patient German population (Schwarz and Hinz, 2001). 63.7 and 69.2 points (means) for global QoL were very similar to our findings.

The relationship between patient-reported global QoL and doctors' judgement were analysed in more detail using various correlational/regressional methods.

**Figure 4:**
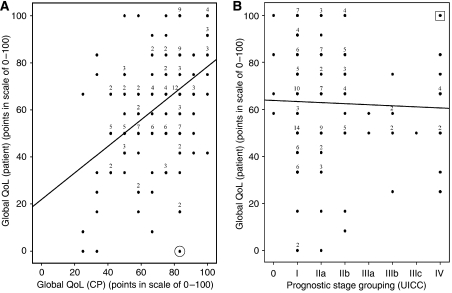
Relationship between assessment of global QoL by the patient herself and by her CP. Comparison with the UICC grouping. For conditions of assessment see Patients and methods. For UICC grading a linear scale was assumed for the statistical model. (**A**) *y*=22+0.56x; Spearman ρ=0.43; *P*(2*α*)<0.01; *n*=158; ⊙ case with the strongest disagreement, for details of this patient see Figure 1. (**B**) *y*=64−0.48x; Spearman ρ=0.006; p(2*α*)=n.s.; *n*=148; 
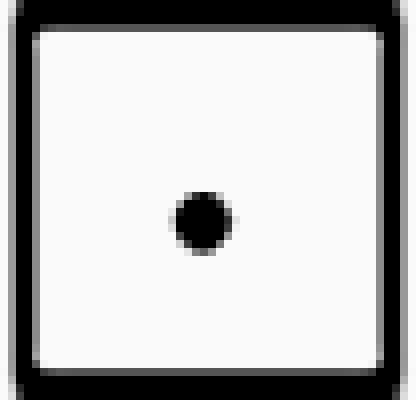
 case with the worst prognosis, but maximum global QoL.

There was significant correlation (Spearman ρ 0.43, *P*<0.001), but the single values were scattered all over the graph (Figure 4A). One exception occurred: very bad values (below 25 points) for global QoL were not assessed by the carefully trained CPs. The case with the strongest disagreement between patient and CP is marked in the lower right corner of Figure 4A:

The patient reported the least favourable overall response (0), whereas doctor's judgement was as high as 83 score points. The QoL profile (Figure 1), demonstrated, in addition to zero global QoL, <50 score point impairments in three specific dimensions: arm symptoms, pain, and emotion.

Doctors presumed that prognosis and QoL were interrelated. This became apparent in a correlation between doctors' judgements of patient global QoL and objective UICC stage, Spearman ρ of −0.28, *P*<0.01 (*n*=147).

However, this was not true when comparing patient-reported global QoL with the UICC prognostic stage (Figure 4B), Spearman ρ of 0.006, *P*=0.94 (*n*=148). Two-thirds of patients with UICC stage IV expressed normal global QoL. One patient scored even a maximum 100 global QoL score points. In contrast, almost half of the UICC I patients (most of whom are expected to survive) demonstrated QoL ⩽50 points (left part of Figure 4B).

The correlation coefficient, however, is just an indicator of a linear relationship, but not one of agreement (in the sense that two judgements are identical). Reliability analysis was performed to answer this question using the intraclass correlation coefficient (Shrout and Ford, 1979) and Cicchetti and Sparrow's (1981) judgement of <0.4, 0.4–0.59, 0.6–0.74 and ⩾0.75 as poor, fair, good and excellent. In the sample of 158 patients the intraclass correlation coefficient was 0.54 (95% C.I. 0.37–0.66), corresponding only to fair reliability.

**Figure 5:**
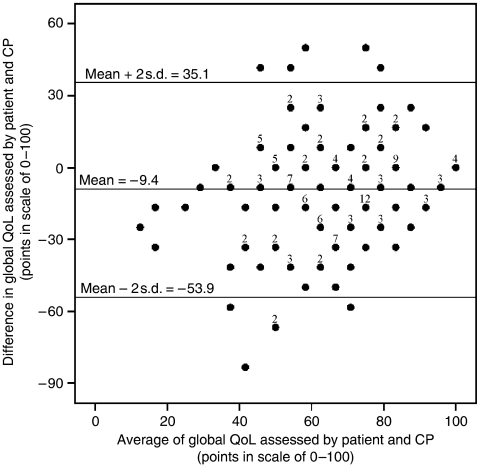
Bland–Altman plot (Bland and Altman, 1986) for agreement analysis between the judgement of the patient and her CP about global QoL. Differences and mean values were calculated for each of the patients. Limits of agreement are the upper and the lower two s.d. values calculated for normal distribution of all differences in the sample (*n*=158).

To elucidate the clinical relevance of disagreement between the judgement of patient and corresponding CP, a Bland-Altman diagram was plotted (Figure 5) (Bland and Altman, 1986). It demonstrated a considerable lack of agreement. First, on average, patient QoL scores were lower than doctors' estimates (mean difference=9.4 score points). Second, data points were scattered across the whole diagram. Hence one judgement could not be replaced by the other (Bland and Altman, 1986), whereas judgements of extreme values of QoL (<40 and >80 score points) remained within the levels of agreement (mean±2 s.d.), in-between values (>40 and <80 score points) showed much more discrepancies, often outside the levels of agreement. These deviations by far exceeded that commonly agreed upon criterion of minimal–clinically–relevant difference of 10 score points (Osoba *et al*, 1998; Norman *et al*, 2003).

**Figure 6:**
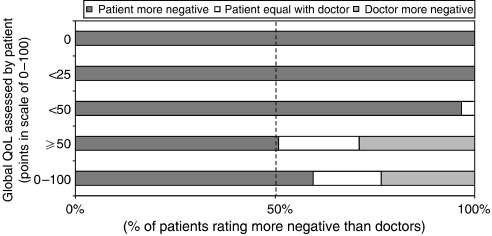
Direction of relative negativity patient/doctor in global QoL depending on decrease of global QoL assessed by the patient.

One aspect of clinical relevance that had not yet been touched by the previous methods was the degree of relative negativity. In other words, how often was the patient or the doctor more negative in judging global QoL? A sensitivity analysis, using five value ranges of patient-reported QoL (0, <25, <50, ⩾50, 0–100) as anchors, was performed (Figure 6). In the normal range (⩾50 points) relative negativity followed largely chance distribution, but below 50 points relative negativity was on the patient's side, that is, patients' self-reports are much more negative than doctors' judgements. Doctors failed completely to recognise very bad QoL (<25 and 0 points).

### Are there empirical reasons supporting the 50-point cutoff criterion in the QoL profile to discriminate between healthy and diseased QoL?

Conceptual reasons (Koller and Lorenz, 2002) led us to suggest a 50-point criterion in the QoL profile to discriminate between ‘healthy’ and ‘diseased’ states in all listed 10 dimensions. Interpretation and reliable threshold levels are major points of discussion. In the literature there is no consensus on a gold standard (FDA, 2006); but are there empirical reasons to support the 50-point criterion?

**Figure 7:**
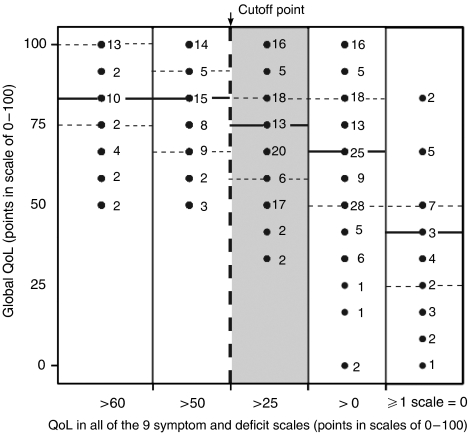
Influence of drop of QoL in single symptom dimensions of QoL on global QoL. Sensitivity analysis with five subgroups: all nine values of the single dimensions >60, >50, >25, >0 or at least one with 0 points. Note that global QoL decreases (reacts) at single items <50 points (between 100 and 26, grey section of the figure). — =median; ....... =interquartile range; cutoff point separating healthy from diseased QoL.

One way of looking at the boundary conditions of decline in global QoL is to investigate threshold levels of specific QoL dimensions and their relations to global QoL (Figure 7). If all values for specific QoL dimensions, such as pain or arm symptoms were >60 or >50 (and therefore in the satisfactory range) (Schwarz and Hinz, 2001), global QoL in none of the patients fell below 50 (left side of Figure 7). However, the turning point was <50 thus supporting the 50 score points as the cutoff between ‘healthy and diseased’. Figure 7 very clearly showed the intimate relations between QoL in single, specific dimensions and global QoL; the negative extreme, zero value in any specific dimension was associated with a dramatic decline in global QoL (right part of Figure 7).

**Figure 8:**
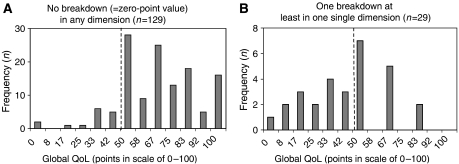
Histograms of global QoL of patients (**A**) either with no very bad value (0-value=worst breakdown) in one of the symptom and deficit scales or (**B**) with at least one very bad value (0-value) in one of the symptom and deficit scales. For reasons of simplicity, there was no differentiation between 0 in one dimension or the other. 50 points=cutoff between healthy and diseased QoL.

This effect became even more apparent in Figure 8A and B: if no zero-point breakdown was observed in the QoL profile, the vast majority of global QoL scores was in the ‘normal’ >50 score point range and only 13 of 129 (10%) patients showed a global QoL<50 (Figure 8A). If, however, at least one single zero-point breakdown was detected, 15 of 29 (52%) patients showed a global QoL<50 (Figure 8B).

## Discussion

Data analysis of the exploratory trial was guided by three questions.

### (1) The selection of patients for implementation by their CPs

It uncovered age discrimination (‘patients over 70 years do not need treatment of their (bad) quality of life!’). Other reasons for the biased selection were proposed by the CPs in this study. They claimed that they see more often younger patients in daily practice than older ones and have therefore selected them just by chance. Older patients are less mobile, and are more likely to have called a CP to their home. Furthermore, many patients came to the CPs early after surgery. This was not the case with older patients (Watermann *et al*, 2005). Finally, younger patients often demonstrated more dramatic cases with many social problems and were therefore selected by the CPs for the QoL study. As the German study demonstrated (Watermann *et al*, 2005), older patients (>70 years) received less chemo- and radiotherapy than younger patients. This has consequences for randomised trials: selection bias must be controlled as older patients are underrepresented in clinical trials and are at risk for higher mortality rates. These problems are now becoming widely recognised and the EORTC has installed a Task Force for the Elderly (Wildiers *et al*, 2007).

Elderly patients should not be excluded from QoL therapy; quite to the contrary they might be the age group that may mostly profit. Therefore, the study protocol for the randomised trial took special care not to exclude elderly patients (Koller *et al*, 2006).

### (2) Disagreement between the patients' and doctors' assessment and judgement of quality of life

This question is not new in QoL research, but remained a matter of persisting concern (Koller *et al*, 1996; Sneeuw and Aaronson, 2002; Groenvold *et al*, 2007). Factors responsible for the disagreement include concreteness, visibility, subjectivity, living arrangements, caring function, close proximity to the patient (Sneeuw and Aaronson, 2002), and also employment of well-validated QoL questionnaires, longitudinal design and sufficient sample sizes (small: *n*<50, definitive: *n*>100). The present exploratory trial differs from those approaches in several ways:

- The situation was routine clinical care (Klinkhammer-Schalke *et al*, 2008), not a scientific setting with its well-known reductionism.
- Patients and doctors used the same Likert-scale-type question to judge patient global QoL. Doctors gave their judgement at the very end of the health status form that included all relevant information on patients' current status of health and treatment and also psychosocial co-fatalities (Troidl *et al*, 1998).
- The doctors were trained in QoL assessment during the implementation procedure, especially by outreach visits (Klinkhammer-Schalke *et al*, 2008). Hence they knew about the subject and what to do.
- Finally, the influence of relatives on QoL (social stigma (Koller *et al*, 1996)) was avoided by patients filling out the questionnaires in a separate room of the CPs. Despite those restrictions against bias, the disagreement remained very large.

The consequences for the subsequent randomised trial were:

- Instead of debating who is right, the patient or the doctor, both sides of the outcome model (Lorenz and Koller, 2002) have to be integrated (Table 1). This is achieved by the multidisciplinary expert team in the QoL system.
- The expert report, however, has to give precise recommendations to the CPs (Figure 1).
- A telephone-based recall system was installed to check whether the CP has used the expert report in his/her decision.

### (3) 50-point cutoff criterion for healthy *vs* diseased QoL

For two reasons (Koller and Lorenz, 2002) a score value of 50 in a range from 0=very bad to 100=very good was regarded as the threshold level for medical intervention (iatrotropy):

1. The EORTC QoL questionnaire items tap into patients' degree of impairment and answers can be given on Likert scales of 1–4 (1=not at all, 4=very much so). Responses of the patient to a symptom item (e.g., pain) can be easily dichotomised with 1 and 2 as the ‘good’ side and 3 and 4 as the ‘bad’ side. Hence, face validity is in favour of the 50-point cutoff criterion (Koller and Lorenz, 2002).
2. According to theories on adaptation level and social comparison (Festinger, 1954) persons generally try to perform slightly better than average. Values under average (50 points) are undesirable; the goal of therapy is to bring patients to over 50 points. Indeed, the normal values for global QoL in the German population were found between 60 and 80 points (Schwarz and Hinz, 2001). Also for pain therapy in a surgery clinic, 50 points on a visual analogue scale from 0 to 100 were taken as the threshold value for intervention (Lempa *et al*, 2000).

Two empirical arguments in favour of the 50-point positivity criterion were found in the exploratory trial.

The first argument came from the negative extreme. If the worst value in one dimension of the QoL profile (0 points) was obtained, global QoL shifted dramatically down to values below 50 points. Global QoL is a summary measure indicating necessity for taking action (iatrotropy) (Wulff and Gotzsche, 2000).

The second argument came from the positive extreme and was derived from sensitivity analysis: how much must QoL decrease in any of nine single dimensions before global QoL as a summary measure reacts with a decline below 50 points? Again, if at least one specific QoL dimension decreased below 50 points then also global QoL showed an effect (Figure 8). If any dimension of QoL decreased to 0 the average value for global QoL fell below 50 points, even including interquartile range. In summary, this relationship between global QoL and any other specific dimension of QoL is in favour of selecting 50 points as a cutoff criterion for all dimensions in the QoL profile (Koller *et al*, 2006).

## Conclusion

A system of QoL diagnosis and therapy was successfully implemented into routine patient care within a Tumor Center. Patients' responses and doctors' judgements are necessary to arrive at a full, clinically meaningful picture of patients' well-being and health status. A theoretically plausible and empirically proven threshold level must be applied to distinguish between satisfactory and critical levels of QoL in various dimensions.

After successful implementation, the QoL system is now ready for exploration by definitive randomised controlled clinical trial (Koller *et al*, 2006).

## Figures and Tables

**Table 1:** Conceptual, methodological and practical prerequisites of the QoL system (Klinkhammer-Schalke *et al*, 2008)

Three component outcome model	Posits three components to assess a patient's ‘true’ end point: (1) objective (e.g., survival); (2) experiential (e.g., mood); and (3) a judgement of clinical relevance, (i.e., which end point is most important)
Threshold value	On a scale from 0 (very bad) to 100 (very good) a value below 50 is regarded as ‘diseased’, because this implies ‘quite a bit’ or ‘very much’ symptom appraisal on the corresponding item.
Quality of life profile	Contains 10 QoL dimensions (derived from EORTC-QLQ-C30+BR23), all scores uniformly scaled from 0 to 100 and displayed in vertical. The graphic presentation allows to spot QoL impairments at a glance.
Health status form	Includes basic information regarding clinical (e.g., tumour stage) and selected non-clinical variables (e.g., co-fatalities) and doctor's overall judgement of patient's QoL at the time patients report on their QoL.
Expert report	Five experts from different disciplines (medicine, psychology) individually diagnosed QoL profiles and the concurrent health status forms, and merged their perspectives into one single report that is structured (1) findings, (2) interpretation, and (3) therapeutic recommendation.
Care pathway	Describes the logical chain of the various elements of primary and follow-up care according to current guidelines, supplemented by QoL assessments and interventions at designated time points.
Quality of life enhancing treatment options	Evidence suggests that the following treatment options have QoL enhancing properties: physiotherapy and lymphatic drainage, pain therapy, psychotherapy, nutrition counseling and physical fitness, social rehabilitation.

**Table 2:** Characteristics of the patients in the implementation sample (*n*=170)

**Attributes of the patients**	**Numbers and rates**
Age (years, *x˜* (range)) (*n*=170)	58 (34–86)
Time after primary treatment (months, *x˜* (i.q. range and range)) (*n*=168)	4 (1–31) and (1–202)
	
*Prognostic stage grouping (UICC,* *n=153)*	
UICC 0	4
UICC I	65
UICC II (II a and b combined)	64
UICC III (III a, b, c combined)	9
UICC IV	11
	
*Receptor status*	
Estrogen positive/negative	137/23
Progesterone positive/negative	126/34
Her2neu positive/negative	35/82
	
*Present phase of therapy*	
Postoperative, BCT/mastectomy	100/63
Chemotherapy positive/negative	20/148
Radiotherapy positive/negative	7/161
Endocrine therapy positive/negative	61/107
	
*Professional status*	
Housewife	46
Working outside the home	100
On pension	24
	
*Family status*	
Married/not married	122/40
Children/no children	137/28

Numbers less than 170 in the sample are due to missing values.
